# Mesenteric cystic lymphangioma in an adult: An unusual case report

**DOI:** 10.1016/j.amsu.2022.103917

**Published:** 2022-06-04

**Authors:** Mohamed Yassine Mabrouk, Oumaima Magouri, Ayoub Madani, Abdelali Guellil, Fatima zahra Rahou, Laila bouzayan, Soufiane Taibi, Tarik Deflaoui, Rachid Jabi, Mohammed Bouziane

**Affiliations:** a Department of General Surgery, Mohamed VI University Hospital, Oujda, Morocco; b Faculty of Medicine and Pharmacy, Laboratory of Anatomy, Microsurgery and Surgery Experimental and Medical Simulation (LAMCESM), Mohammed 1st University, Oujda, Morocco; c Department of Pathology, Mohammed VI University Hospital, Oujda, Morocco

**Keywords:** Cystic lymphangioma, Mesenteric tumor, Surgical excision, Case report

## Abstract

**Introduction:**

Cystic lymphangioma is a benign malformation tumor of the lymphatic system. Its location is variable, and mesenteric localization remains extremely rare.

**Case presentation:**

We describe a rare case of cystic lymphangioma of the mesentery in a 26 years old woman. The diagnosis was suspected following an abdominopelvic computed tomography (CT) and magnetic resonance imaging (MRI), showing a large polylobulated cyst in contact with the stomach, the tail of the pancreas, the spleen, and the antero-external cortex of the left kidney. The patient underwent laparoscopic surgery with a pericystectomy. Pathological examination confirmed the diagnosis of cystic lymphangioma of the mesentery. The patient's postoperative recovery was uneventful. After a Follow up of one year after surgery, there was no evidence of recurrence.

**Clinical discussion:**

Cystic lymphangioma of the mesentery is a benign malformation tumor of the lymphatic system. Its clinical aspects are very polymorphic; the diagnosis is evoked by radiological imaging but requires pathological confirmation. Surgery is the gold standard in the management of this pathology.

**Conclusion:**

We highlight the importance of radical surgical resection to avoid Cystic lymphangioma complications and minimize the recurrence risk.

## Highlights

• The mesenteric location of cystic lymphangioma remains extremely rare.
• Preoperative diagnosis is usually challenging
• The final diagnosis is made upon pathology study.
• Complete en-bloc resection is the gold standard treatement.

## 1 Introduction

Cystic lymphangiomas are rare vascular malformation conjunctive tumors corresponding to sequestration of lymphatic tissue secondary to an embryological developmental anomaly of the lymphatic system [1]. They are rare benign tumors that are mostly seen in children. In adults, they represent 7% of abdominal cysts [2]. Their clinical manifestations are not very suggestive and are not specific depending on the location and the size of the cyst [3]. The diagnosis is evoked by radiological imaging which includes computed tomography (CT), and magnetic resonance imaging (MRI) but only pathological examination can provide a certain diagnosis [4].

Herein, we report a rare case of cystic lymphangioma of the mesentery in an adult patient which presents a particular interest in its diagnosis, treatment, and prognosis.

We have followed the Surgical Case Report (SCARE) 2020 guidelines in reporting this work [5].

## 2 Case report

A 26 years-old women patient was referred by a family physician to our department for management of a left hypochondrium pain evolving for two months without fever, vomiting, nausea, or any other symptom of gastrointestinal obstruction, her surgical and medical history was unremarkable.

The clinical examination finds a soft, tender abdomen with accentuated pain in the left hypochondrium and epigastrium with no abdominal palpable mass.

As part of the etiological evaluation, routine labs including blood tests, and hydatid serology were within normal ranges. The tumor markers (ACE, and CA19-9) were normal.

**Fig. 1:**
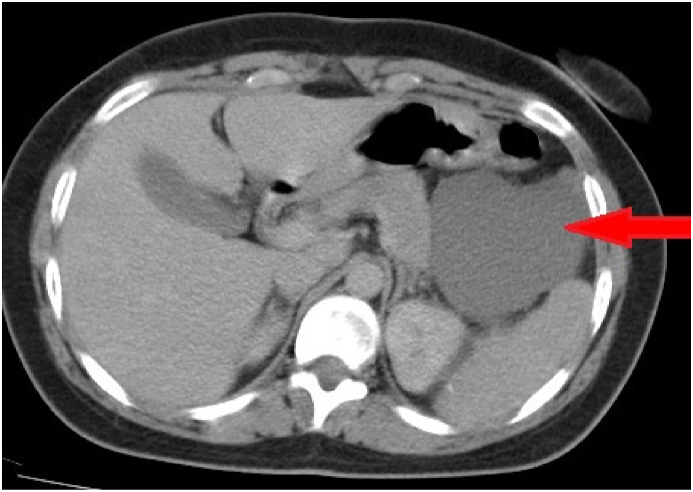
Abdominal CT scan showing a cystic mass in contact with the stomach, the tail of the pancreas, the spleen, and the Antero external cortex of the left kidney.

**Fig. 2:**
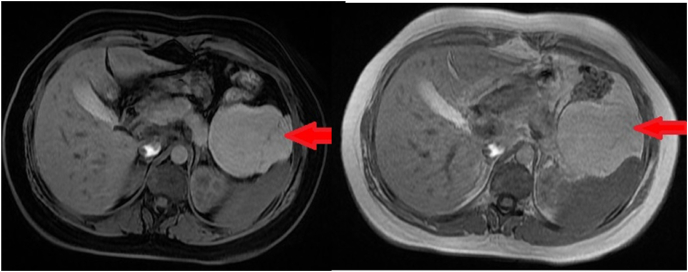
Abdominal MRI showing an intraperitoneal cystic mass located at the level of the left hypochondrium presenting hypointensity in the T1 weighted and hyperintensity at T2 weighted with some fine partitions.

An abdominal CT scan was performed showing a large polylobulated cyst measuring 76*62 mm with an intracystic tissue component in contact with the stomach, the tail of the pancreas, the spleen, and the antero-external cortex of the left kidney (Fig. 1). The abdominal MRI objectified an intraperitoneal cystic abdominal mass located in the left hypochondrium, measuring 80*70 mm presenting hypointensity in the T1 weighted and hyperintensity at T2 weighted. (Fig. 2).

**Fig. 3:**
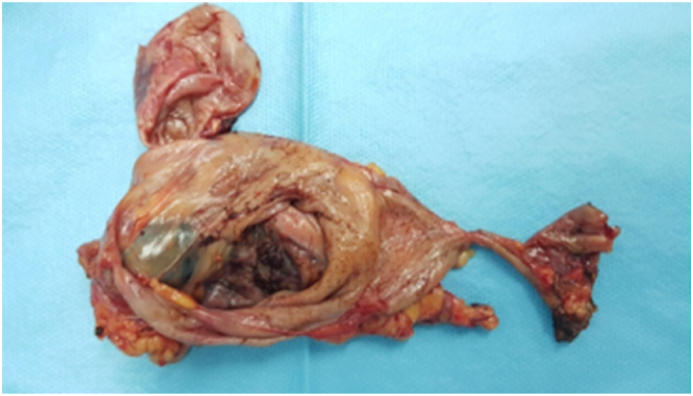
Image showing the resected mass.

She underwent laparoscopic surgery under general anesthesia; intraoperative exploration finds cystic mass infiltrating the lower face of the pancreas, the spleen with intimate contact with the transverse colon, and the left colonic angle. We decided to realize a pericystectomy after a meticulous release of the mass from its attachments (Fig. 3). The intervention was accomplished by a professor of general surgery.

Postoperatively, Our patient received antibiotics based on Amoxicillin clavulanic acid (1g every 8hours), analgesia (Nefopam 20mg every 6 hours), and prophylactic heparin therapy (4000 UI per day) were administered.

**Fig. 4:**
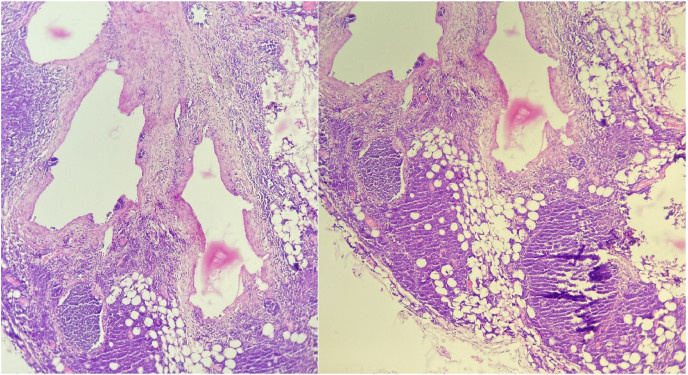
The microphotographic analysis showing cystic lymphatic cavities of variable size, lined with endothelial cells resting on a fibrous wall with an inflammatory lymphocytic infiltrate creating lymphoid nodules; Hematoxylin and eosin (H&E) stain x400.

Pathological examination founds cystic lymphatic cavities of variable size, lined with endothelial cells resting on a fibrous wall with an inflammatory lymphocytic infiltrate creating lymphoid nodules (Fig. 4).

The patient's postoperative recovery was unremarkable; she was discharged from the hospital on the fifth postoperative day.

After a Follow up of one year after surgery, there was no evidence of recurrence.

## 3 Discussion

Cystic lymphangiomas are benign tumors of the lymphatic vessels, which are mostly observed in children, they are rare in adults, and they are generally located in the head, neck, and axillary region; abdominal locations are rarer [6]. Cystic lymphangiomas can affect all organs, except the central nervous system, which central nervous system is devoid of lymphatic [7]. In its mesenteric location, the incidence is estimated at 1/100000 in adults and 1/20000 in children [8], with a female-to-male ratio of around 1 in adulthood, while in childhood this ratio is either similar or slightly predominant in boys [9].

The clinical presentation of cystic lymphangiomas is variable. When the lesion is symptomatic, the clinical signs are related to the volume of the tumor or a complication, ranging from an often asymptomatic mass in adults to acute abdominal pain or even complications such as rupture, infection, intracystic hemorrhage, occlusion, torsion, compression or infiltration of vital structures.

Differential diagnoses include a wide range of cystic intraabdominal lesions, such as mesenteric cysts, abdominal lymphomas, secondary metastases from an unknown primary tumor, tuberculosis, hydatid disease, small bowel adenocarcinomas, and rare mesenteric tumors, including desmoid tumors, schwannomas, smooth muscle tumors, sarcomas, cystic mesotheliomas, lymphangiosarcomas, and lymphangiomas with myxoid degeneration [10].

No sign is specific, and it is the radiological imaging that will orient the diagnosis [11].

Abdominal ultrasounds show a well-limited liquid tumor composed of multilocular anechoic cysts with thin walls without calcification. However, the contents may become echogenic when there is an intracystic hemorrhage, or even contain some calcifications [12].

The CT scan is the gold standard for the diagnosis, allowing to study the density of the tumor, to evaluate the density of the tumor and its relationship with the neighboring organs, and to differentiate retroperitoneal lymphangioma from intraperitoneal lymphangioma. Magnetic resonance imaging is more specific for the content of the cyst showing a precise diagnosis and appreciating very well the perivascular extension of the lesion [12].

Diagnostic certainty is provided by pathological analysis of the tumor. Macroscopically, cystic lymphangioma can be single or polycystic, with oligo-macrocytic, micro-polycystic, and mixed forms. Microscopically, typical diagnostic features of the diagnosis are dilated lymphatic vessels, lined with flattened endothelial cells without atypia, with abundant lymphoid tissue. We can also find in their wall smooth muscle cells and foamy cells containing lipid material [13].

In the absence of treatment, cystic lymphangiomas progressively increase in volume in the abdominal cavity and lead to mechanical complications by compression of the neighborhood organs. It can also be complicated by rupture, infection, or intracystic hemorrhage [14]. Malignant transformation is exceptional [15].

Complete surgical resection is the gold standard in the treatment of abdominal cystic lymphangiomas [16]. Complete excision should be attempted, taking care to remain as conservative as possible for the other organs, due to the benign nature of the lymphangioma [17]. A large exeresis is an absolute requirement to avoid recurrence which is estimated to be 40%. Complete removal of the cyst can be facilitated by intraoperative aspiration of the contents. If the cyst infiltrates nearby organs such as the spleen, intestine, and pancreas, the cyst itself and these organs must be resected [18]. For unresectable cystic lymphangiomas, percutaneous sclerosing injections with OK-432, Ethibloc®, and doxycycline have been tried and have been shown effective in the treatment of superficial and deep cystic lymphangiomas but these results have not been evaluated in the long term [19]. A Targeted therapy, which is acting specifically on tumor cells sparing the normal Cells represents a new perspective of the treatment of mesenteric cystic lymphangioma involving a specific lymphatic marker including PRox-1, VEGFR-3, PDGFR-b, D2-40, particularly for those who show aggressive or recurrent behavior [20].

## 4 Conclusion

Intra-abdominal cystic lymphangioma is a rare benign tumor with clinical and radiological polymorphism. The preoperative and even intraoperative diagnosis is not always easy in adults. The final diagnosis is made upon pathological examination of the surgical specimen. The treatment of choice is complete surgical resection of the tumor.

## Funding

No sources of funding to our research has been needed.

## Ethical approval

No ethical approval necessary.

## Consent

Written informed consent was obtained from the patient for publication of this case report and accompanying images. A copy of the written consent is available for review by the Editor-in Chief of this journal on request.

## Sources of funding

The author(s) received no financial support for the research, authorship and/or publication of this article.

## Author contribution

**Dr Mabrouk Mohamed Yassine:** Have written the article, prepared the patient for surgery and participated in the surgery. **Dr Oumaima Magouri:** Interpretation of histological data. **Dr Ayoub Madani, Dr Abdelali Guellil**: participated in the surgery. **Dr Rahou Fatima zahra, Dr Laila bouzayan, Dr Soufiane Taibi, Dr Tarik Deflaoui:** Contributed for diagnose and treatment of the patient. **Pr Jabi Rachid:** supervised the writing of manuscript. **Pr Bouziane Mohammed** (oncology surgery professor): have supervised the writing of the paper, and has been the leader surgeon of the case.

## Registration of research studies

Our paper is a case report; no registration was done for it.

## Guarantor

Mabrouk Mohamed Yassine.

## Declaration of competing interest

The authors declared no potential conflicts of interests with respect to research, authorship and/or publication of the article.
